# Posterior endpoint determination of the lumbar pedicle central axis on the anterior–posterior fluoroscopic image for pedicle screw insertion

**DOI:** 10.1038/s41598-024-57349-8

**Published:** 2024-04-23

**Authors:** Jun Zhang, Jiawei Xu, Chenyang Xu, Youzhuan Xie

**Affiliations:** 1 Shanghai Key Laboratory of Orthopaedic Implant, Department of Orthopaedic Surgery, Ninth People’s Hospital, Shanghai Jiaotong University School of Medicine, Shanghai, China; 2 Department of Orthopaedic Surgery, Qingpu Branch of Zhongshan Hospital Affiliated to Fudan University, Shanghai, China; 3 Department of Orthopaedic Surgery, Quanzhou Taiwanese Investment Zone Hospital, Fujian, China

**Keywords:** Lumbar spine, Pedicle central axis, Fluoroscopy, Computer-aided design, Three-dimensional printing, Entry point, Anatomy, Medical research, Neurology

## Abstract

The transpedicular procedure has been widely used in spinal surgery. The determination of the best entry point is the key to perform a successful transpedicular procedure. Various techniques have been used to determine this point, but the results are variable. This study was carried out to determine the posterior endpoint of the lumbar pedicle central axis on the standard anterior–posterior (AP) fluoroscopic images. Computer-aided design technology was used to determine the pedicle central axis and the posterior endpoint of the pedicle central axis on the posterior aspect of the vertebra. The standard AP fluoroscopic image of the lumbar vertebral models by three-dimensional printing was achieved. The endpoint projection on the AP fluoroscopic image was determined in reference to the pedicle cortex projection by the measurements of the angle and distance on the established X–Y coordinate system of the radiologic image. The projection of posterior endpoint of the lumbar pedicle central axis were found to be superior to the X-axis of the established X–Y coordinate system and was located on the pedicle cortex projection on the standard AP fluoroscopic image of the vertebra. The projection point was distributed in different sectors in the coordinate system. It was located superior to the X-axis by 18° to 26° at L1, while they were located superior to the X-axis by 12° to 14° at L2 to L5. The projections of posterior endpoints of the lumbar pedicle central axis were located in different positions on the standard AP fluoroscopic image of the vertebra. The determination method of the projection point was helpful for selecting an entry point for a transpedicular procedure with a fluoroscopic technique.

## Introduction

The transpedicular procedure has been widely used in spinal surgery. Posterior transpedicular screw fixation has been most commonly used for management of an unstable lumbar spine caused by trauma, degenerative conditions, scoliosis, and extensive laminectomies^1–4^. With the development of minimally invasive spine surgery, procedures such as vertebroplasty, kyphoplasty, and percutaneous pedicle screw fixation have become the main techniques for managing traumatic vertebral fractures, metastatic vertebral body tumors, and osteoporotic vertebral fractures^5–11^.

The key to performing a successful transpedicular procedure is determining the optimal entry point. Penetration should ideally occur along the central axis of the pedicle, incorporating the maximal available transverse and sagittal pedicle diameters^12^. Moreover, biomechanical studies have shown that a pedicle screw placed along the central axis of the pedicle can achieve the most stable fixation point and has the greatest clinical effect^13^. Physical and radiological anatomy studies have provided various methods for determining the entry point. However, the data from these studies vary greatly^14–17^.

In this study, the computer-aided design and three-dimensional (3D) printing were used to determine the posterior endpoint of the lumbar pedicle central axis on the anterior–posterior (AP) fluoroscopic image of vertebra. The study was aimed to provide improved guidance for selecting the entry point of the guide needle in a transpedicular procedure with a fluoroscopic method.

## Materials and methods

The computed tomography (CT) scans of 30 normal adults undergoing examination were investigated. People with lumbar fractures, spondylolisthesis, tumors, infection, or degenerative deformities were excluded. The digital imaging and communication in medicine (DICOM) format data were downloaded from the GE Advantage CT workstation (Little Chalfont, UK).

This study was approved by the ethics committee of Shanghai Ninth People's Hospital. Informed consent was obtained from all subjects and/or their legal guardian(s). All experiments were performed in accordance with relevant guidelines and regulations.

### Three-dimensional (3D) lumbar reconstruction

CT data of lumbar spines were used to construct 3D images by Mimics software (version 16.0; Materialize, Leuven, Belgium). After reconstruction, the coronal, transverse, sagittal, and 3D lumbar images were simultaneously displayed.

### Pedicle isthmus central axis determination and 3D printing of a computer-designed lumbar model

**Figure 1:**
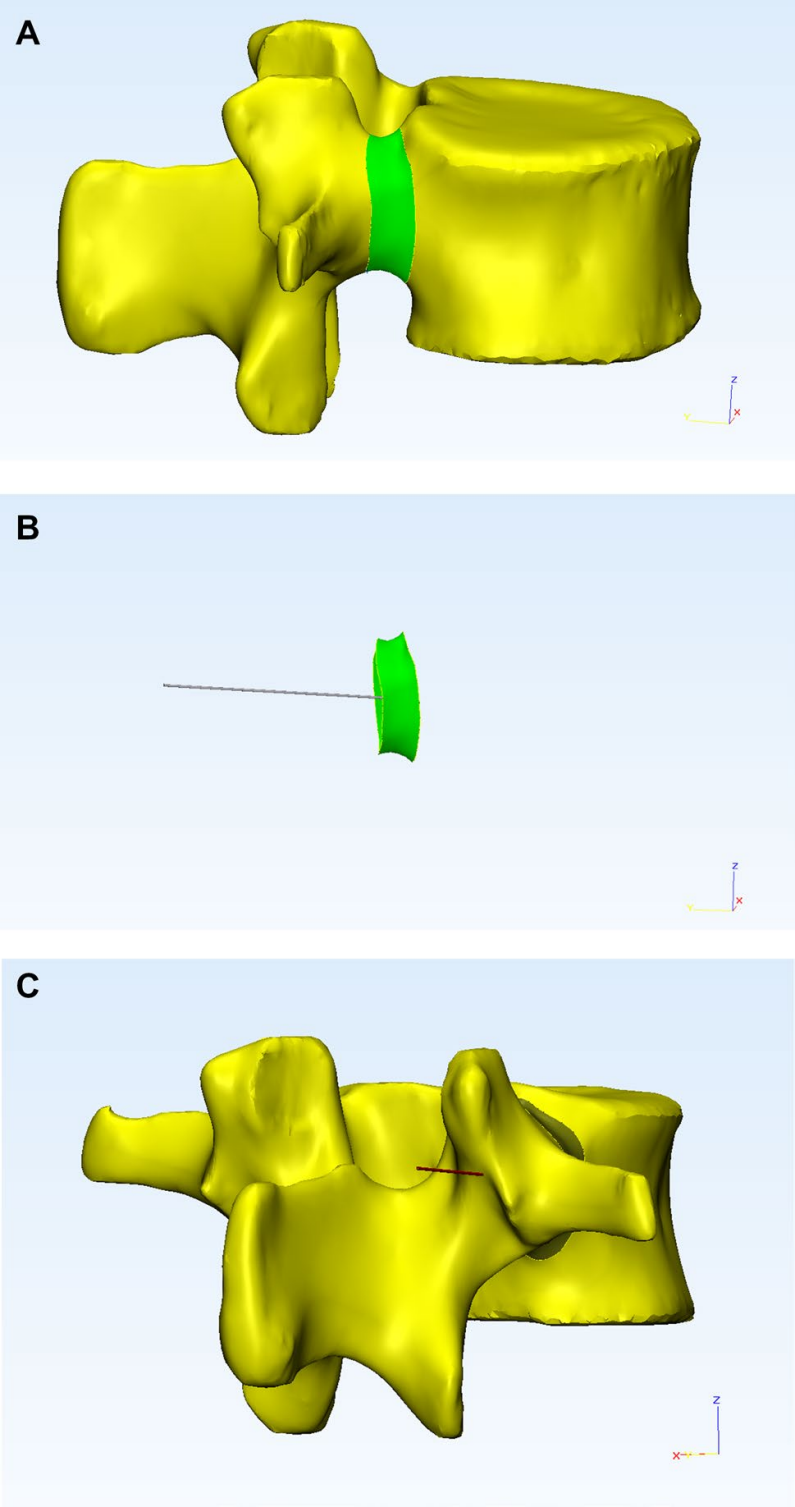
(**A**) The isthmus of the pedicle was isolated from the intact pedicle on the reconstructed vertebra with the 3-matic software. (**B**) Pedicle isthmus axis was calculated by the *Create Line* operation function of the 3-matic software. (**C**) The intersection between the pedicle isthmus axis and the posterior surface of the vertebra was determined to be the posterior endpoint of pedicle central axis.

In this study, the central axis of the pedicle was defined as the pedicle isthmus axis, which is the narrowest section of the pedicle. 3D reconstruction of the digital lumbar data was imported into the 3-matic software (version 8.0; Materialize, Leuven, Belgium). The pedicle isthmus was isolated from the intact pedicle by the function of software(Fig. 1A). And the isthmus axis was then obtained by another function of the same software (Fig. 1B). The 3D model of pedicle isthmus axis was exported in STL format.

Subsequently, the 3D reconstructed pedicle isthmus axis model was imported into lumbar 3D reconstruction images in Mimics. The intersection of the extended pedicle isthmus axis and the posterior aspect of the vertebra was the posterior endpoint of pedicle central axis (Fig. 1C). Then the intersection point was marked in this lumbar model. Finally, the data of the lumbar spine model marked with the intersection point were exported in an STL file. Based on the file, nylon powder was laser-fused layer-by-layer to create a 3D structural lumbar model by the Selective Laser Sintering (SLS) technology (FS251P, Hunan Farsoon High-Technology Co., Ltd., Changsha, China).

### Posterior endpoint determination of lumbar pedicle central axis on AP fluoroscopic images

**Figure 2:**
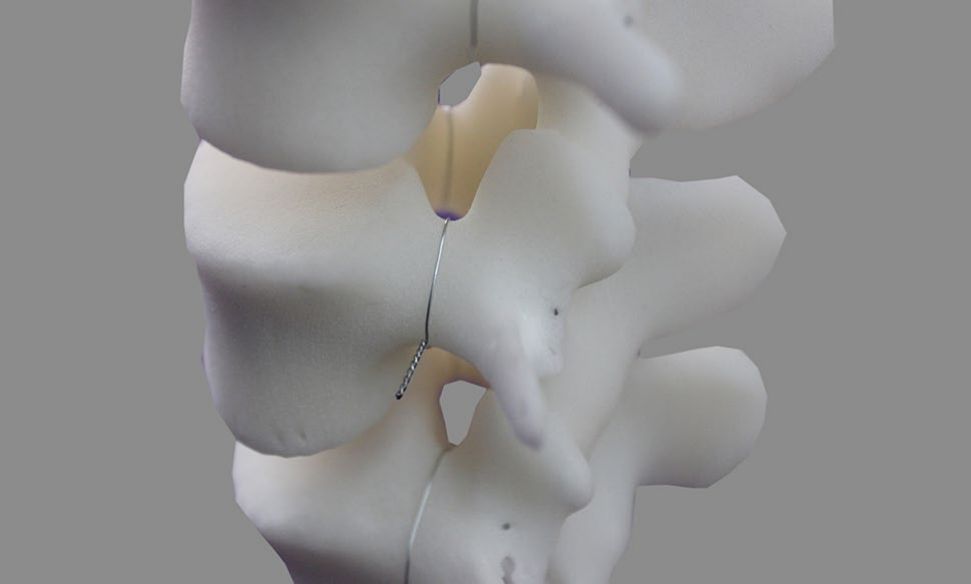
A fine malleable wire was wounded tightly against the outer border of the pedicle isthmus. The wire pellet was embedded into the marked endpoint on the model.

As the pedicle isthmus margin of the plastic model was not clearly distinguishable compared with the cadaver specimen on the fluoroscopic image, the malleable wire was wounded (0.31-mm diameter) tightly around the outer surface of the pedicle isthmus of the model in order to improve measurement accuracy. The wire halo was corresponded to the pedicle cortex projection under the X-ray fluoroscopy^18^. Furthermore, the wire pellets (0.9 mm in diameter, 2 mm in length) were embedded into the marked posterior endpoint of pedicle central axis on the lumbar model (Fig. 2).

**Figure 3:**
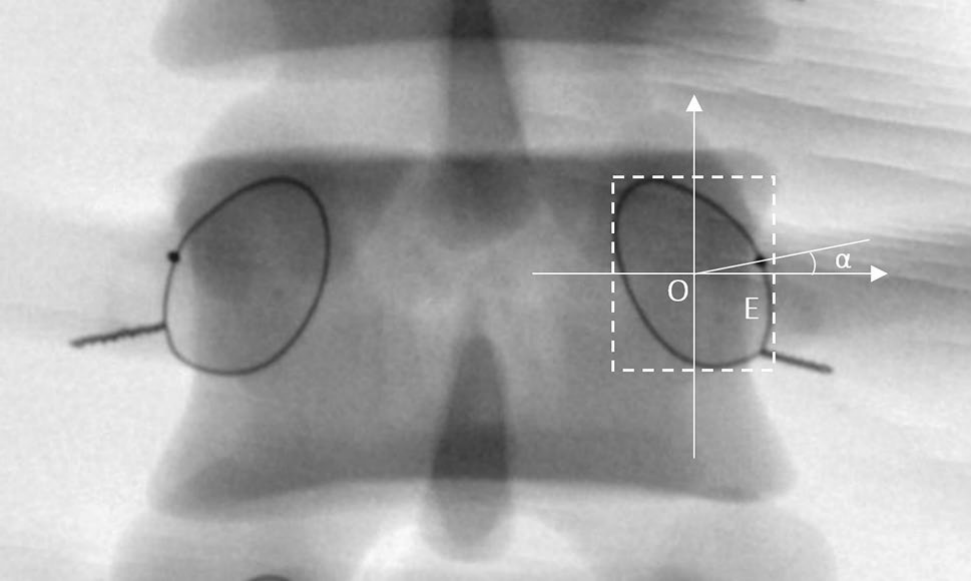
The 'one line' sign was seen on the standard anterior–posterior fluoroscopic image. The spinous process was found in the middle of the two pedicles. Dashed rectangle was surrounded by four lines, i.e., two lines parallel to the endplate through the top and bottom of the wire halo and two lines vertical to the endplate through the medial and lateral borders of the wire halo. In the coordinate system, the origin of the coordinate was positioned at the center of the rectangle. The X-axis toward outward was positive and the Y-axis toward cranial was positive. The α angle was measured between a line from the wire pellet projection (E point) to the origin (O point) and the X-axis.

The standard AP fluoroscopic images of each vertebra, in which the endplate appeared as the “one line” sign and the spinous process was found in the middle of the two pedicles, were obtained by adjusting the spot angle of the C-arm. A rectangle closely around each wire halo was created by drawing two lines (cephalic and caudal) parallel to the endplates and two lines (medial and lateral) vertical to the endplate with the help of a Digimizer (MedCalc Software, Ostend, Belgium). An X–Y coordinate system with the origin located at the center of the rectangle was created. The angle between the line from the wire pellet projection to the origin and the X-axis was measured. The angle value was defined as positive when the wire pellet projection was located superior to the X-axis, and vice versa (Fig. 3). The distance from the wire pellet projection to the inner margin of the wire halo was measured. If the pellet projection was located within the wire halo, the distance value was defined as negative, and vice versa.

### Data collection

Specialized and standardized training was provided to the individuals involved in this study, ensuring that data collection adhered to established norms. Cases were randomly assigned to different measurers. The final results underwent review and assessment by two attending clinical physicians.

### Statistical analysis

The Graphpad Prism6 software for Windows (La Jolla, CA, USA) was used for statistical analysis. Every parameter was reported as the means and standard deviation. The paired *t-*test was used to compare the difference between men and women and the difference between the left and the right pedicles for all the data collected. One-way ANOVA was used to analyze the difference among each segmental parameter respectively. P < 0.01 was considered as statistically significant.

## Results

The lumbar CT data were collected from 15 male and 15 female people with the mean age of 32.7 ± 6.8 years (range, 20–45 years). The average height was 168.9 ± 8.2 cm(range, 158–182 cm). A total of 300 pedicles were reconstructed, and 60 pedicle central axial posterior endpoints were measured at each segment. There were no statistically significant differences (P > 0.05, paired *t-*test) between the data from men and women or between the left and right pedicles at each segment(Tables 1 and 2).

**Table 1:** The angle (degree) between the line from the projection point to the origin and the X-axis in different sides and gender (n = 15).

	Side^a^		Gender^b^	
	Left	Right	Male	Female
L1	22.39 ± 16.32	21.43 ± 14.19	24.33 ± 17.98	19.49 ± 11.53
L2	9.06 ± 5.65	10.35 ± 6.18	9.58 ± 6.97	9.83 ± 4.72
L3	10.51 ± 4.24	11.37 ± 4.00	11.29 ± 4.98	10.59 ± 3.04
L4	10.55 ± 9.35	12.47 ± 7.62	11.08 ± 10.55	11.93 ± 5.98
L5	10.18 ± 6.35	11.84 ± 5.77	10.88 ± 7.44	11.14 ± 4.44

^a^P > 0.05, paired *t-*test.

^b^P > 0.05, independent samples t-test.

**Table 2:** The distance (mm) from the projection point to the inner margin of the wire halo in different sides and gender (n = 15).

	Side^a^		Gender^b^	
	Left	Right	Male	Female
L1	− 0.87 ± 0.53	− 0.77 ± 0.53	− 0.86 ± 0.64	− 0.78 ± 0.38
L2	− 0.90 ± 0.52	− 0.77 ± 0.49	− 0.89 ± 0.59	− 0.78 ± 0.39
L3	− 0.31 ± 0.65	− 0.32 ± 0.63	− 0.42 ± 0.76	− 0.22 ± 0.48
L4	− 0.70 ± 0.66	− 0.78 ± 0.64	− 0.73 ± 0.74	− 0.75 ± 0.54
L5	− 0.42 ± 0.76	− 0.61 ± 0.81	− 0.46 ± 0.94	− 0.57 ± 0.61

^a^P > 0.05, paired *t-*test.

^b^P > 0.05, independent samples t-test.

**Figure 4:**
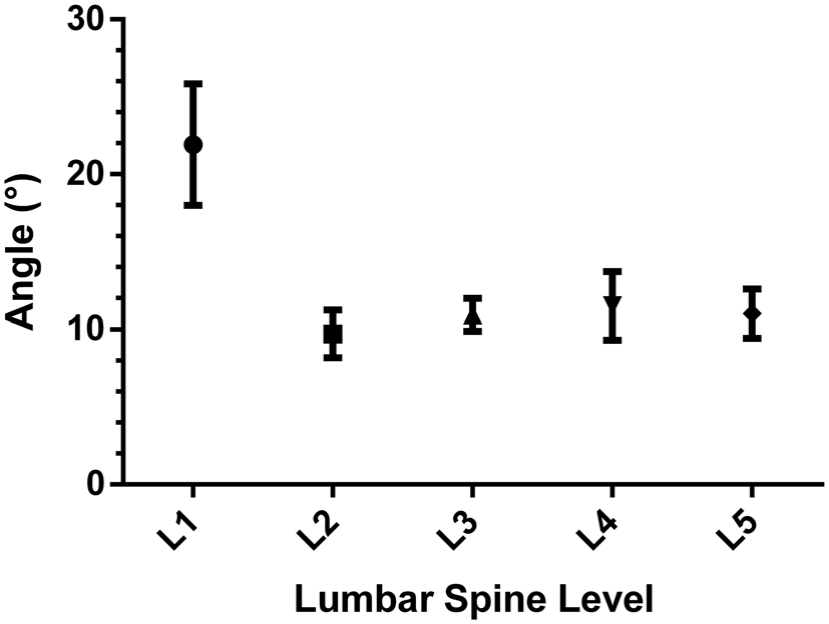
95% Confidence Interval (95% CI) of the angle between the line from the projection point to the origin and the X-axis.

The posterior endpoints from L1 to L5 were found to be superior to the X-axis of the coordinate system. The angles between the line from the projection point to the origin of the coordinate system and the X-axis were positive values (Table 3). The angles were 9.71 ± 5.90°at L2, 10.94 ± 4.11°at L3, 11.51 ± 8.51° at L4, and 11.01 ± 6.08° at L5. There was no statistically significant difference among the angles (P > 0.05, One-way ANOVA). However, the angle at L1 was 24.91 ± 15.17°, which was significantly different from the angle at other lumbar segments (P < 0.0001, One-way ANOVA) (Fig. 4).

**Table 3:** The angle (degree) between the line from the projection point to the origin and the X-axis (n = 15).

	L1*	L2^#^	L3^#^	L4^#^	L5^#^
Mean	21.91	9.71	10.94	11.51	11.01
Minimum	− 9.25	0.0	2.31	− 8.46	− 5.32
Maximum	67.31	23.26	22.10	40.07	27.91
Standard deviation	15.17	5.90	4.11	8.51	6.08
95% confidence interval	17.99–25.83	8.18–11.23	9.88–12.00	9.31–13.70	9.44–12.58

*P < 0.01, ^#^P > 0.05, One-way ANOVA (Bonferroni-adjusted p-value).

**Figure 5:**
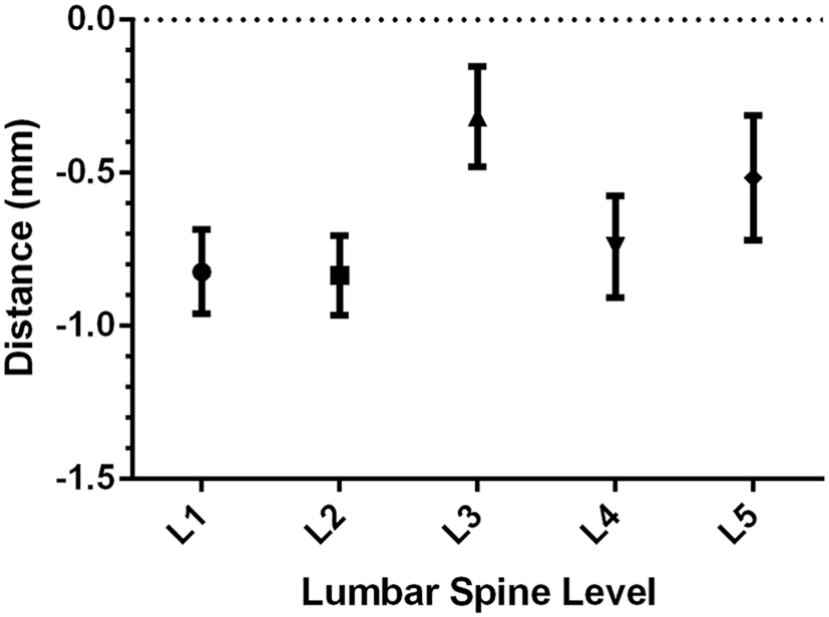
95% Confidence Interval (95% CI) of the distance from the projection point to the inner margin of the wire halo.

The posterior endpoints of the central pedicle axis from L1 to L5 were located within the wire halo. The distance from the projection point to the inner margin of the wire halo was negative value. The distance was − 0.82 ± 0.53 mm at L1, − 0.84 ± 0.50 mm at L2, − 0.32 ± 0.64 mm at L3, − 0.74 ± 0.64 mm at L4, and − 0.52 ± 0.79 mm at L5 (Table 4, Fig. 5). There was no significant difference among these values (P > 0.05, One-way ANOVA).

**Table 4:** The distance (mm) from the projection point to the inner margin of the wire halo (n = 15).

	L1*	L2*	L3*	L4*	L5*
Mean	− 0.82	− 0.84	− 0.32	− 0.74	− 0.52
Minimum	− 2.16	− 2.36	− 1.99	− 2.78	− 2.45
Maximum	0.0	0.45	1.08	0.24	1.39
Standard deviation	0.53	0.50	0.64	0.65	0.79
95% confidence interval	− 0.96 to − 0.69	− 0.96 to − 0.71	− 0.48 to − 0.15	− 0.91 to − 0.57	− 0.72 to − 0.31

*P > 0.05, One-way ANOVA (Bonferroni-adjusted p-value).

## Discussion

In order to decrease the risk of surgical complications such as nerve, vascular, and spinal cord injury, a transpedicular procedure should be performed along the axis of the pedicle, taking full advantage of the transverse and sagittal diameters^12^. The best entry point for a lumbar transpedicular procedure should be the projection point of posterior endpoint of the pedicle central axis on the fluoroscopic image.

In previous studies^19,20^, anatomical landmarks have been used to determine the exact location of the projection point. Hou et al.^19^ proposed zone concepts to define the projection points on the posterior surface of the lamina. They concluded that the projection points were above the midline of the transverse process and were consistently near the midline of the transverse process from L1 to L4; however, at L5 the projection point was located on the mid-transverse process and 4.9 mm away from the most lateral border of the superior facet. While Ebraheim et al.^20^ concluded that the projection points were averaged 3.9 mm, 2.8 mm, and 1.4 mm superior to the midline of the transverse process for L1, L2, and L3, and averaged 0.5 mm and 1.5 mm inferior to the midline of the transverse process for L4 and L5, respectively.

Not only do these data vary greatly, it is also of note that normal anatomical landmarks may be lacking in individuals with anatomical abnormalities, such as spinal deformity, fracture, and facet joint hypertrophy. In addition, with the development of minimally invasive techniques, the use of percutaneous procedures has become increasingly popular^21–25^. In these situations, fluoroscopic guidance should be used to determine of the best entry point.

The best entry point should be posterior endpoint of the pedicle central axis. The position of this point on the AP fluoroscopic image should be determined. Before the establishment of this position, the central axis of the pedicle should first be defined. Ebraheim et al.^20^ and Wang et al.^26^ defined the pedicle central axis as the intersection of two planes, i.e., the transverse plane through the midline of the transverse process, and the sagittal plane through the midline of the vertical pedicle diameter. In the current study, the central axis of the pedicle was defined as the pedicle isthmus axis, and the posterior endpoint was defined as the point where the pedicle isthmus axis intersected with the posterior surface of the vertebra. The pedicles did not have an accurate geometrical central axis because of their complicated cylinder structures. However, the pedicle isthmus was the narrowest part of the pedicle in the transverse plane. Its structure was easy to follow and a transpedicular procedure could be successfully performed if only the cortical bone of the isthmus was not penetrated^27^. Thus, the authors considered the isthmus axis as the pedicle central axis from a surgical point of view. Moreover, the determination of the pedicle axis and the position of the posterior endpoint by the computer-aided design was more precise than the one determined by manually searching in the cadaveric specimens in the previous studies^20,26^, and this would lead to more precise results.

However, Andrew et al.^28^ reported the anatomical relationship between the accessory process of the lumbar spine and the pedicle screw entry point. They thought the accessory process is a consistent and reliable landmark to guide pedicle screw entry point. This may be a potential reference anatomical structure.

**Figure 6:**
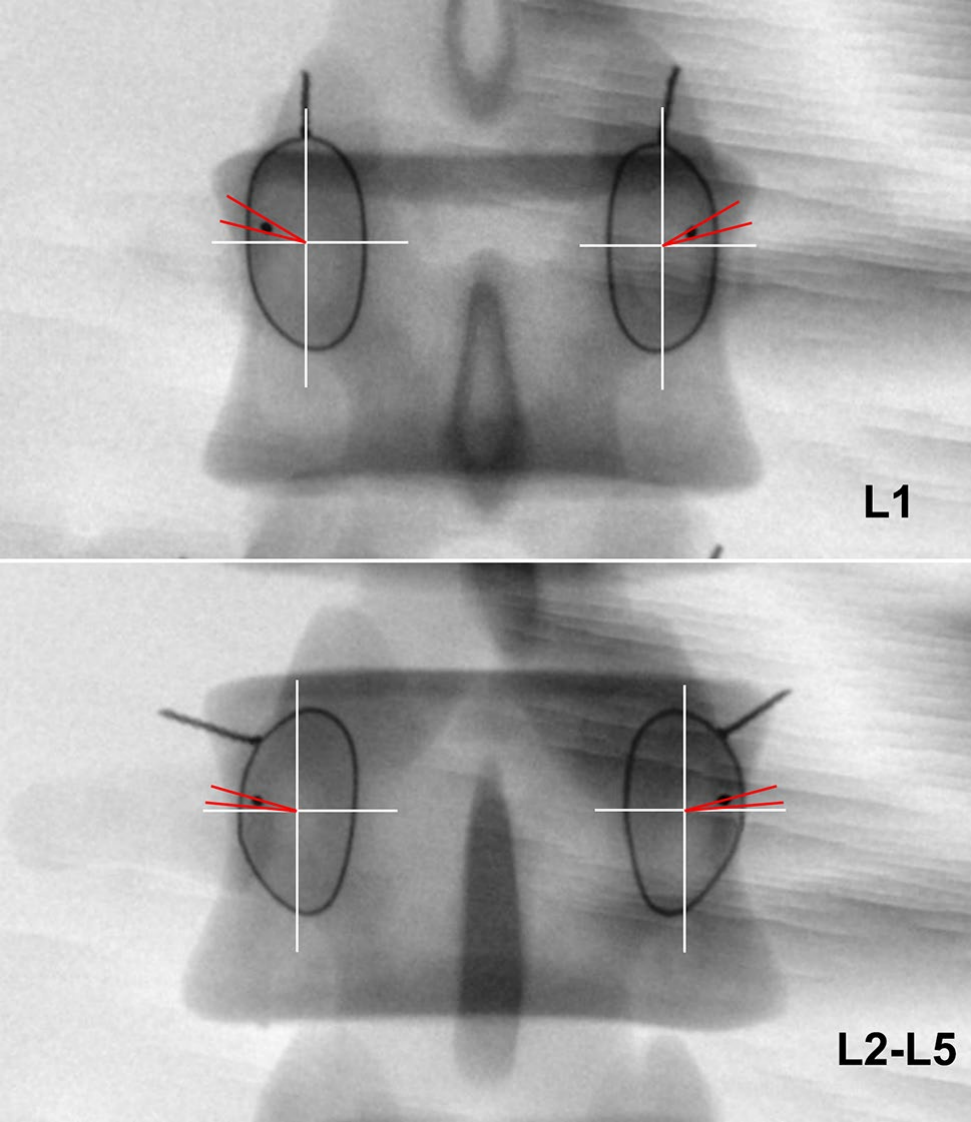
At L1 the projection point was located superior to the X-axis by 18° to 26° (demonstrated by the red lines). At L2-L5 the projection points were located superior to the X-axis by 12° to 14° (demonstrated by the red lines). The projection points of lumbar were located on the pedicle cortex projection.

This study showed that the posterior endpoint of lumbar pedicle central axis were distributed across different sectors for each segment on the established X–Y coordinate system on the standard AP fluoroscopic image of the vertebra. The point at L1 was located superior to the X-axis by 18° to 26°, the projection points at L2 ~ L5 were located superior to the X-axis by 12° to 14° (Fig. 6). While Wang et al.^26^ demonstrated that the projection point was located at the 9 o’clock to 11 o’clock position of the left pedicle projection (1 o’clock to 3 o’clock position of the right pedicle projection), this study provided a more pricise location of the entry point to guide the surgery. These results would therefore decrease the risk of surgical complications. The distance from the projection point to the inner margin of the wire halo was found to be less than 1 mm on the inside of the wire halo at all levels. Therefore, the posterior endpoints of the lumbar pedicle central axis were located on the pedicle cortex projection, as the cortical image of the pedicle was considered to have a maximum density of 1–3 mm within the pedicle isthmus wire halo^18^. The finding was consistent with the Wang et al.'s^26^.

In the current study, the computer-aided design and 3D printing technology were applied to reproduce a total of 30 lumbar spine (300 pedicles) models. These 3D printed models were more cost-effective and more easily available compared with the cadaveric specimens. They were the promising substitutes for cadaveric specimens for some large-scale orthopedic and radiologic research. Furthermore, The malleable wire around the outer surface of the pedicle isthmus was useful to improve measurement accuracy. The effectiveness of this method has been verified by Robertson et al.^18^.

The present study has some limitations. The posterior endpoint determination was a little difficult at the L5 level in clinical percutaneous procedures because the lateral border of the L5 pedicle was superimposed on the image of the lateral border of the L5 body owing to its large pedicle transverse angle^18^.

This study provided a new perspective on the selection of the optimal entry point for pedicle screw placement. However, it is essential to note that medial breech is an important complication that cannot be ignored during the pedicle screw implantation process^29^. The optimal angle for pedicle screw insertion is also a subject that requires further investigation, representing a limitation of this study.

## Conclusion

In conclusion, with the help of the 3D computer design and printing technique, the projections of posterior endpoints of the lumbar pedicle central axis were found to be located in different positions on the standard frontal fluoroscopic image of the vertebra. The results provided an improved guidance for selecting the entry point of the guide wire in a transpedicular procedure with a fluoroscopic method.

## Data Availability

The data that support the findings of this study are available from Shanghai Ninth People's Hospital but restrictions apply to the availability of these data, which were used under license for the current study, and so are not publicly available. Data are however available from the authors upon reasonable request and with permission of Shanghai Ninth People's Hospital. For further information, please contact gxjxzj518@163.com.
